# MDM2 inhibitor ameliorates cisplatin‐induced nephropathy via NFκΒ signal inhibition

**DOI:** 10.1002/prp2.450

**Published:** 2018-12-13

**Authors:** Tomoyuki Fujikura, Hideo Yasuda, Takamasa Iwakura, Takayuki Tsuji, Hans‐J. Anders

**Affiliations:** ^1^ First Department of Medicine Hamamatsu University School of Medicine Hamamatsu, Shizuoka Japan; ^2^ Renal Division Medizinische Klinik und Poliklinik IV Klinikum der Universität München LMU München Munich Germany

**Keywords:** acute kidney injury, Cisplatin, Murine double minute 2 (MDM2)

## Abstract

Cisplatin is a platinum‐containing chemotherapeutic drug, which is widely used and highly effective. While effective against tumors, its use is limited by severe side effects such as nephrotoxicity and bone marrow suppression. Murine double minute 2 (MDM2) is the E3 ubiquitin ligase of the tumor suppressor gene, p53, and inhibition of MDM2 can suppress tumor cell growth. However, independent of p53, MDM2 acts as a co‐transcription factor for nuclear factor‐κB (NFκB), whose signaling can be involved in cisplatin‐induced tubular injury. We therefore examined the effects of MDM2 inhibitor on cisplatin cytotoxicity. In order to induce acute kidney injury and to investigate MDM2 inhibitory effects, we injected cisplatin into rats with or without the MDM2 inhibitor, DS‐5272, and analyzed kidney physiology/histology and NFκB signaling. Serum creatinine was significantly lower in the DS‐5272 group than in the vehicle group on day 3 (0.55 ± 0.069 vs 0.70 ± 0.072 mg/dL, *P* < 0.05). DS‐5272 also significantly decreased kidney injury molecule‐1 (KIM‐1) expression, improved tubular injury, and decreased apoptotic cells. Western blotting showed that cisplatin increased NFκB phosphorylation in kidneys, which was significantly suppressed by DS‐5272. In vitro, we treated HEK 293 cells with cisplatin, in the absence or presence of DS‐5272, and examined cytotoxicity and NFκB transcriptional activity. DS‐5272 co‐treatment reduced both cisplatin‐induced cell death and NFκB transcriptional activity. Collectively, these findings suggest that DS‐5272 can ameliorate cisplatin nephrotoxicity via NFκB signal inhibition.

**Abbreviations**
LDH: lactate dehydrogenase
PAS: periodic acid‐Schiff
TUNEL: terminal deoxynucleotidyl transferase dUTP nick end labeling

## 1 INTRODUCTION

Cisplatin (cis‐diamminedichloro‐platinum (II)) is a platinum‐containing cancer therapy drug, that is widely used and highly effective. It is used for the treatment of solid tumors (eg, nonsmall cell lung cancer, squamous carcinoma of the head and neck, ovarian cancer and bladder cancer). Although cisplatin is effective against various tumors, it is limited by severe side effects such as nephrotoxicity and bone marrow suppression. The prevalence of nephrotoxicity is approximately one in three,^1^ with some patients experiencing progressive kidney injury and irreversible renal failure. Therefore, effective treatments, which can prevent cisplatin nephrotoxicity and do not enhance tumor growth are required.

Murine double minute 2 (MDM2) is the E3 ubiquitin ligase of the tumor suppressor gene, p53, and acts as a co‐transcription factor for nuclear factor‐κB (NFκB).^2^ MDM2 inhibitor exhibits no toxicity against normal tissue, but it can function as a cancer therapy drug that assembles p53 and induces cancer cell death.^3^ Therefore, combination therapy of MDM2 inhibitor and cisplatin may have synergistic effects.^4^, ^5^, ^6^ However, MDM2 inhibitor has been reported to suppress cell apoptosis induced by cisplatin in vitro.^7^ The inhibition of p53 or NFκB signaling has also been shown to improve acute kidney injury induced by cisplatin.^8^, ^9^, ^10^, ^11^ Therefore, the effects of MDM2 inhibitor on cisplatin‐exposed normal tubular and cancerous cells may be different.

We have already reported that MDM2 inhibitor has protective effects in acute ischemic kidney injury 12 and crescentic glomerulonephritis 13 in vivo and that the efficacy of MDM2 inhibitor is associated with NFκB inhibition, but not that of p53 signaling. We, therefore, hypothesized that MDM2 inhibitor has protective effects on cisplatin nephropathy by blocking NFκB signal transduction. To confirm this hypothesis, we induced nephropathy in vivo or tubular‐cell death in vitro with cisplatin and analyzed the effects of MDM2 inhibitor on this toxicity and on NFκB signaling.

## 2 MATERIALS AND METHODS

### 2.1 Animal studies

Male Sprague‐Dawley rats weighing 200‐250 g (SLC Co., Shizuoka, Japan) were used for the induction of acute kidney injury, because male rats have been shown to be more sensitive than female rats.^14^ The rats were housed individually in wire mesh cages (12‐hours light‐dark cycle) and provided standard rat chow (Rodent Lab Diet EQ 5L37: SLC Co., Shizuoka, Japan) and water ad libitum under specific‐pathogen‐free conditions. The experimental protocol was approved by the Ethics Review Committee for Animal Experimentation of the Hamamatsu University School of Medicine.

The rats, which were randomly allocated, received either saline vehicle or 5 mg/kg cisplatin (provided by Nihon Kayaku Co. Ltd., Tokyo, Japan) intravenously at day 0 in order to induce acute kidney injury. The MDM2 inhibitor, (6R)‐7‐[(4R)‐1‐{[(5R,6S)‐5‐(4‐chloro‐3‐fluorophenyl)‐6‐ (6‐chloropyridin‐3‐yl)‐3‐isopropyl‐6‐methyl‐5,6‐ dihydroimidazo[2,1‐b][1,3]thiazol‐2‐yl]carbonyl}‐4‐fluoro‐L‐ prolyl]‐6‐ethyl‐4,7‐diazaspiro[2.5]octane (DS‐5272), was provided by Daiichi Sankyou Co. Ltd. (Tokyo, Japan). This inhibitor was developed by Miyazaki et al and its chemical structure was shown previously.^15^ Each treatment group had been subdivided to receive either 10 mg/kg DS‐5272 or vehicle intraperitoneally 24 hours before and just after the cisplatin injection. On days 0, 1, 3, 5, and 10, the rats were anesthetized intraperitoneally with ketamine (75 mg/kg) and xylazine (10 mg/kg) and were euthanized by intraperitoneal pentobarbiturate overdose (200 mg/kg), the blood and kidneys were then collected for further investigation. Since we had preliminary data that rats cisplatin‐induced nephropathy was constant between individuals, we used group sizes of n = 4‐5, specifically n = 4 for cisplatin (−)/DS‐5272 (−), n = 5 for cisplatin (−)/DS‐5272 (+), n = 5 for cisplatin (+)/DS‐5272 (−), and n = 5 for cisplatin (+)/DS‐5272 (+) at each time point.

### 2.2 Measurement of creatinine and blood cells counts

Serum creatinine and blood cells counts were measured by enzymatic assays and flow cytometry, respectively (Falco SD, Kyoto, Japan).

### 2.3 Histological evaluation

The kidneys were flushed briefly with phosphate‐buffered saline via the abdominal artery in order to remove erythrocytes that hinder clear examination of kidney histology. The flushed kidneys were fixed with 4% paraformaldehyde and embedded in paraffin for periodic acid‐Schiff (PAS) staining. Tubular injury scores were assessed as the percentage of injured tubules in the corticomedullary junction that showed necrosis, brush border loss, and tubular dilation, as follows: 0, none; 1, 1%‐10%; 2, 11%‐25%; 3, 26%‐45%; 4, 46%‐75%; 5, 76%‐100%. Dead tubular cells were detected by terminal deoxynucleotidyl transferase dUTP nick end labeling (TUNEL) technique using the ApopTag^®^Plus in Situ Apoptosis Detection Kit (Merk Millipore, Darmstadt, Germany)^16^. Histological analyses were performed blindly.

### 2.4 Western blotting

Protein from kidney tissues was extracted with RIPA buffer (Sigma‐Aldrich, Hamburg, Germany) in the presence of Protease and Phosphatase Inhibitor Mini Tablets (Thermo Fisher, Rockford, IL, USA). Equal amounts of protein were loaded onto sodium dodecyl sulfate polyacrylamide gels for electrophoresis and western blotting. The blots were probed with the following primary antibodies at 4°C overnight: anti‐KIM1, anti‐MDM2 (Abcam, Cambridge, UK), anti‐phospho‐MDM2, anti‐p53, anti‐phospho‐p53, anti‐NFκβ, anti‐phospho‐NFκβ, anti‐Iκb, anti‐phospo‐Iκb and β‐actin (Cell Signaling Technology, Danvers, MA, USA). After washing the primary antibody, the membranes were incubated with peroxidase‐conjugated secondary antibody. The bands were visualized by enhanced chemiluminescence (GE Healthcare, Little Chalfont, UK).

### 2.5 In vitro *cell assay*

In order to analyze further the mechanism of tubular cells cytotoxicity in vitro, we utilized the immortalized human tubular cell line, HEK 293, which was kindly provided by Drs. Kitagawa and Niida (Hamamatsu University School of Medicine, Shizuoka, Japan). The cells were maintained for each experiment in Modified Eagle's medium supplemented with 10% fetal bovine serum. For the cytoprotection assay, the cells were exposed to cisplatin (0‐200 μmol/L) with or without DS‐5272 for 24 hours. Cytotoxicity was then measured using a Cytotoxicity Detection Kit (LDH), according to the manufacturer's instructions (Roche, Basel, Switzerland). For detection of NFκB p65‐specific transcriptional activity, the cells were exposed to cisplatin (0 or 100 μmol/L) with or without DS‐5272 for 12 hours, followed by extraction of nuclear protein using a Nuclear Extraction Kit (Abcam). The NFκB p65‐specific transcriptional activity of each nuclear extract was determined using an NFκB p65 Transcription Factor Assay Kit (Abcam) according to the manufacturer's instructions. Each of the above in vitro experiment was performed for five times.

### 2.6 Statistical analysis

All values are expressed as means ± SD. Differences among groups of more than two were examined for statistical significance by one‐way ANOVA with post‐hoc Tukey's multiple comparison test; differences between two groups were examined by unpaired t tests (Prism 7, GraphPad Software, San Diego, CA, USA).

## 3 RESULTS

### 3.1 Renal MDM2 expression increased after cisplatin injection

**Figure 1:**
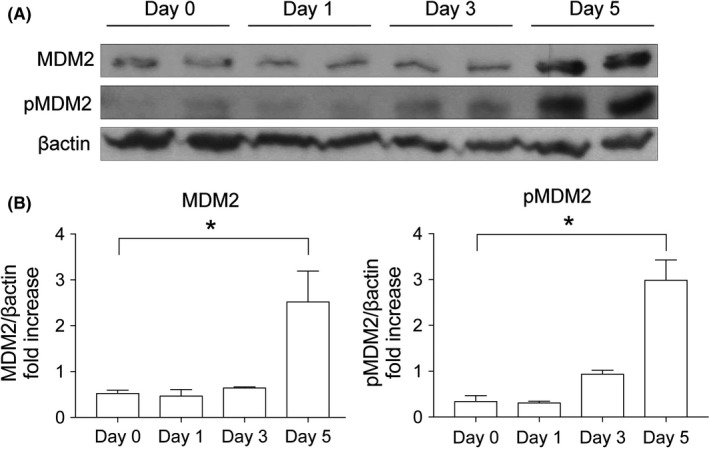
MDM2 expression after Cisplatin injection. Rats were injected with cisplatin (5 mg/kg) and euthanized on Days 0, 1, 3 or 5 (n = 5). (A) MDM2 and phospho‐MDM2 (pMDM2), and β‐actin levels (representative western blots). (B) Densitometric analyses were performed and plotted. *P < 0.05 vs Day 0

We previously confirmed that MDM2 mRNA is expressed in the kidney as well as in the lung, colon, urinary bladder and brain.^12^ Immunostaining of healthy kidneys also revealed MDM2 protein expression in the proximal and distal tubules,^12^ which are targets of cisplatin nephrotoxicity. We explored the expression and phosphorylation of MDM2 by western blotting before and after cisplatin injection. Phospho‐MDM2 started to increase 3 days after cisplatin injection and both MDM2 and phospho‐MDM2 were increased significantly by day 5 (Figure 1A,B). However, on days 1 and 3, both MDM2 and phospho‐MDM2 were not significantly increased. Therefore, MDM2 expression levels were not associated with the early injury phase of cisplatin nephropathy.

### 3.2 DS‐5272 ameliorated cisplatin nephropathy

Since Nutlin3a (20 mg/kg); an MDM2 inhibitor, had been shown to ameliorated kidney injury by ischemia‐reperfusion 12 or crescentic glomerulonephritis,^13^ we anticipated that MDM2 inhibitors would also have some protective effects on cisplatin nephropathy. DS‐5272, the MDM2 inhibitor used in this study, is known to have 4‐to‐5‐fold high activity than Nutlin‐3a to inhibit the growth of cancer cells in vitro. We, therefore, treated the rats simultaneously with DS‐5272 (5,10, 25 or 50 mg/kg) and cisplatin, which resulted in mortalities of 0%, 0%, 35%‐50%, and 100%, respectively, by day 5. Next, we tested administrations regimens of DS‐5272 treatment (i.e, 5 or 10 mg/kg; daily, starting 24 hours before cisplatin injection; for 1, 2, or 3 days) on cisplatin nephropathy. The only protocol that had a protective effect on renal dysfunction induced by cisplatin was two injections of 10 mg/kg DS‐5272 (data not shown), which was used for subsequent experiments in this study.

**Figure 2:**
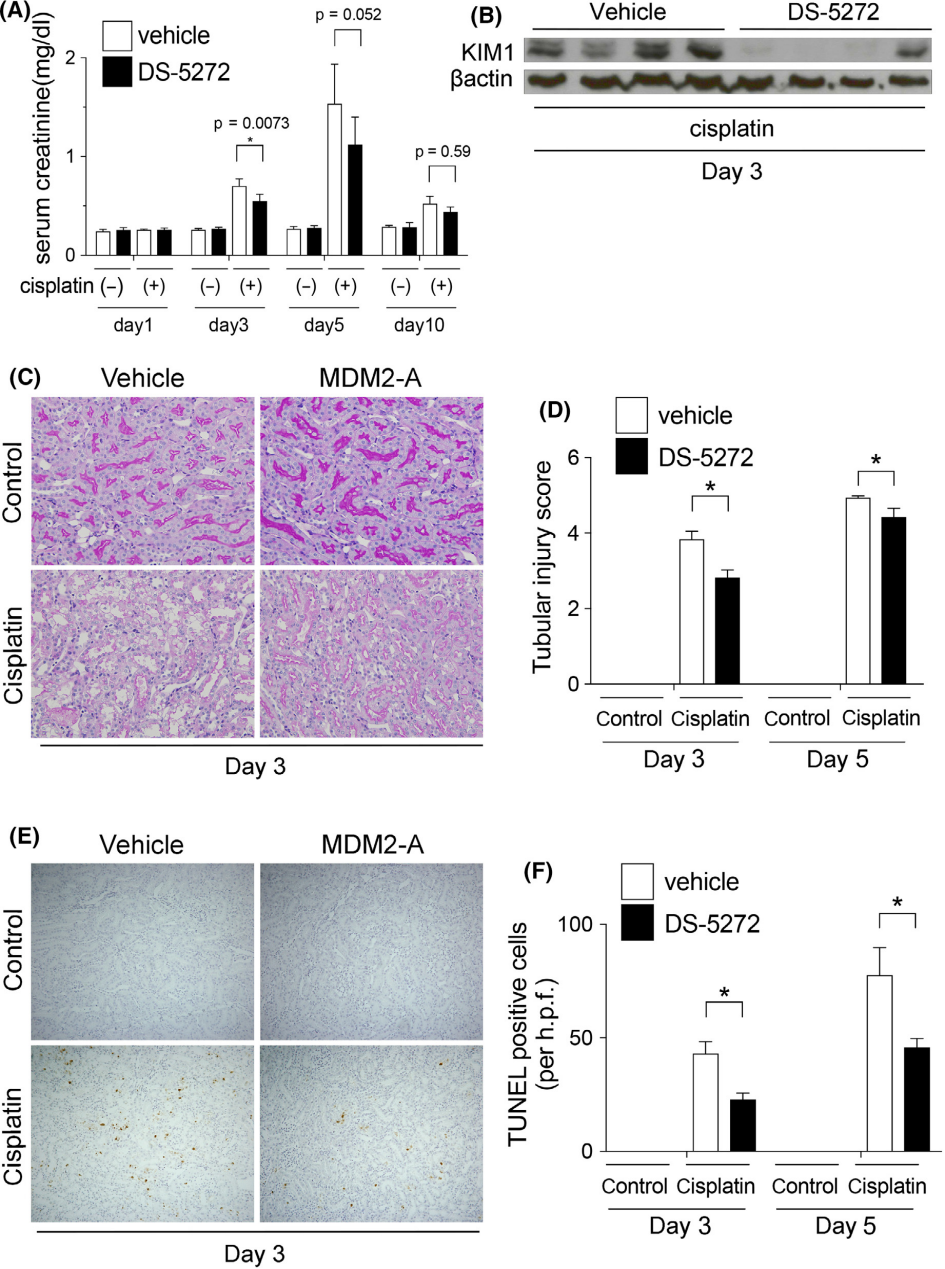
DS‐5272 ameliorates Cisplatin nephropathy. Rats were injected with cisplatin (5 mg/kg) with or without MDM2 inhibitor DS‐5272 (10 mg/kg) (cisplatin (−)/DS‐5272 (−), n = 4; cisplatin (−)/DS‐5272 (+), n = 5; cisplatin (+)/DS‐5272 (−), n = 5; cisplatin (+)/DS‐5272 (+), n = 5). (A) Serum creatinine levels were determined on Days 1, 3, 5 and 10. *P < 0.05 cisplatin (+)/DS‐5272 (−) vs cisplatin (+)/DS‐5272 (+). (B) KIM‐1 expression was determined by western blotting on Day 3. (C) Tubular injuries were visualized by periodic acid‐Schiff (PAS) staining on Day 3 and (D) tubular injury scores were quantified on Days 3 and 5 and plotted. *P < 0.05 cisplatin (+)/DS‐5272 (−) vs cisplatin (+)/DS‐5272 (+). (E) Tubular cell death was analyzed by TUNEL staining on Day 3. (F) TUNEL‐positive cells were quantified on Days 3 and 5 and plotted. *P < 0.05 cisplatin (+)/DS‐5272 (−) vs cisplatin (+)/DS‐5272 (+). All images shown are representative of at least 10 determinations

DS‐5272 significantly decreased the levels of serum creatinine (Figure 2A) and kidney injury molecule‐1 (KIM1) protein, an injury marker of tubules, (Figure 2B) by day 3. PAS staining also confirmed that there was less tubular injury in the MDM2 inhibition group (eg, brush border loss, tubular necrosis, and tubular dilation) than in the vehicle group (ie, cisplatin only; Figure 2C,D). As both necrosis and apoptosis contribute to tubular cell death in cisplatin nephropathy,^17^, ^18^ we performed TUNEL staining that is capable of detecting both apoptotic and necrotic cells.^19^ DS‐5272 significantly decreased TUNEL positive staining at days 3 and 5 (Figure 2E,F). Thus, DS‐5272 ameliorated cisplatin nephropathy.

### 3.3 MDM2 and p53 expressions are not altered at baseline

**Figure 3:**
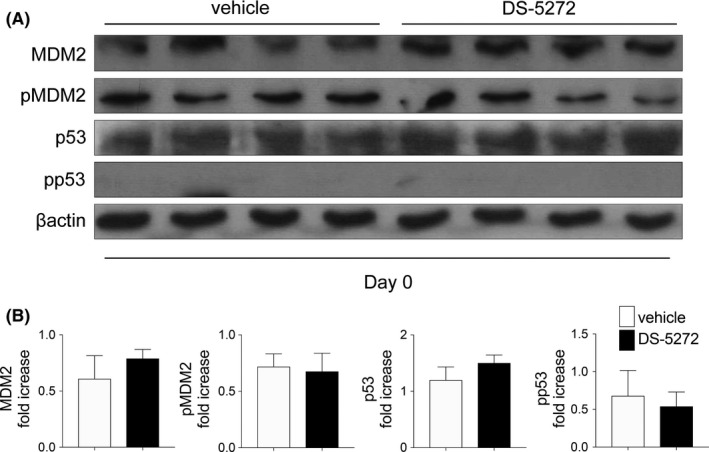
Levels of MDM2, p53 and their phosphorylated forms. (A) Rats were injected with or without MDM2 inhibitor DS‐5272 (10 mg/kg) on Day 1 and protein expression was determined by western blotting on Day 0, just before cisplatin injection. Representative blots are shown. (B) Densitometric analyses were carried out and plots of the quantified data are shown. No statistical differences were determined (Vehicles; n = 4, DS‐5272; n = 5) *P < 0.05

The protein level of MDM2 and phosphorylation of MDM2 started to increase 5 days after cisplatin injection (Figure 1), but the effect of DS‐5272 had already occurred by day 3 (Figure 2). To elucidate whether MDM2 signal inhibition itself contribute to the protective effect, we assessed the expression of MDM2 protein and its target protein, p53, on day 0, just before cisplatin injection, but after DS5272 injection. The protein levels and phosphorylation of both MDM2 and p53 were not changed at day 0 (Figure 3).

### 3.4 Protective effect via NFκB signal inhibition

**Figure 4:**
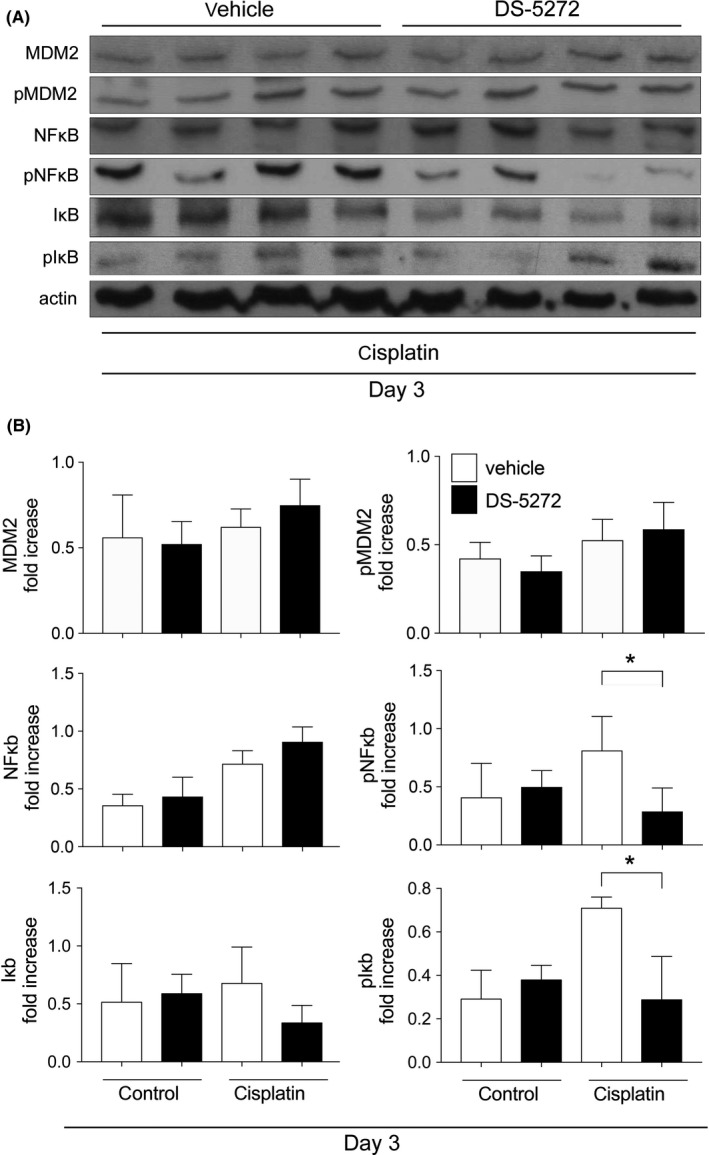
MDM2, NFκB, and Iκb protein and phosphorylation level on Day 3. Rats were injected with cisplatin (5 mg/kg) or saline, with or without MDM2 inhibitor DS‐5272 (10 mg/kg) (cisplatin (−)/DS‐5272 (−); n = 4, cisplatin (−)/DS‐5272 (+); n = 5, cisplatin (+)/DS‐5272 (−); n = 5, cisplatin (+)/DS‐5272 (+); n = 5) (A) Representative western blots and (B) densitometric analyses are shown. *P < 0.05 cisplatin (+)/DS‐5272 (−) vs cisplatin (+)/DS‐5272 (+)

On day 3, neither MDM2 nor phospho‐MDM2 level was changed with DS‐5272 treatment. (Figure 4A). Because Nutlin‐3a was previously shown to ameliorate ischemia‐reperfusion 12 and crescentic glomerulonephritis 13 kidney injury via NFκB signal inhibition, we next investigated NFκB signaling with or without DS‐5272. Although NFκB expressions in the DS‐5272 group were similar to that in the vehicle group, DS‐5272 treatment significantly decreased NFκB phosphorylation compared with that of the vehicle group (Figure 4A,B). DS‐5272 also reduced the IκB level slightly, compared with that of the vehicle group, and IκB phosphorylation was significantly decreased with DS‐5272 treatment (Figure 4A,B). Considering these results collectively, DS‐5272 inhibited NFκB activation.

### 3.5 DS‐5272 inhibits cisplatin‐induced cell death via NFκB signal inhibition

**Figure 5:**
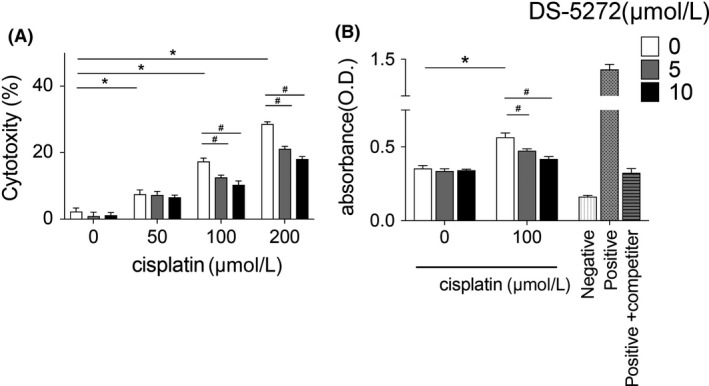
Protective effect of DS‐5272 and NFκB signaling in vitro. HEK 293 cells were cultured with various concentration of cisplatin, with or without DS‐5272. (A) Cytotoxicity was analyzed by lactate dehydrogenase (LDH) release assay 24 hours after cisplatin treatment. (B) NFκB p65 transcriptional activity was analyzed by NFκB p65 Transcription Factor Assay Kit 12 hours after cisplatin treatment. Negative: negative control (without any nuclear extract), positive: positive control (provided in Kit), positive+competitor: positive control and NFκB p65 specific competitor (both were provided in Kit) Each assay was performed five times. *P < 0.05 cisplatin 0 μmol/L vs cisplatin 50, 100, or 200 μmol/L; #*P* < 0.05 DS‐5272 0 μmol/L vs 5 or 10 μmol/L

To investigate the protective effects of DS‐5272 on tubular cells, we treated HEK 293 cells with cisplatin in the absence or presence of DS‐5272. Cisplatin‐induced cell death in a concentration‐dependent manner either with or without DS‐5272 (Figure 5A). However, DS‐5272 significantly and concentration‐dependently protected against cisplatin‐induced cell death (Figure 5A). Next, to explore further whether NFκB signal inhibition contributes to the cytoprotective effects of DS‐5272, we investigated NFκB transcriptional activity. Cisplatin treatment alone increased NFκB transcriptional activity, but DS‐5272 significantly decreased this cisplatin‐induced NFκB activity (Figure 5B). These studies suggested that DS‐5272 inhibits cisplatin‐induced tubular cell death via NFκB signal inhibition.

### 3.6 DS‐5272 does not affect bone marrow suppression after cisplatin injection

**Figure 6:**
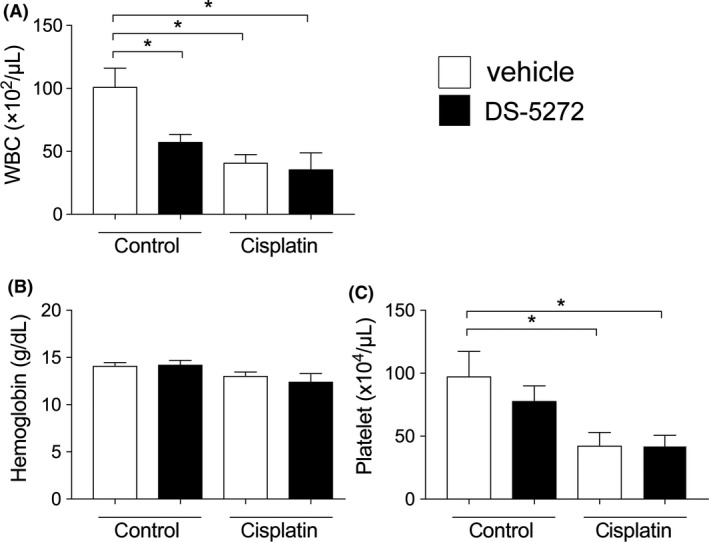
Blood parameters 10 days after cisplatin injection. Rats were injected with cisplatin (5 mg/kg) with or without MDM2 inhibitor DS‐5272 (10 mg/kg). (cisplatin (−)/DS‐5272 (−); n = 4, cisplatin (−)/DS‐5272 (+); n = 5, cisplatin (+)/DS‐5272 (−); n = 5, cisplatin (+)/DS‐5272 (+); n = 5). (A) White blood cell counts, (B) hemoglobin levels and (C) platelet counts were measured 10 days after cisplatin injection. *P < 0.05 vs cisplatin (−)/DS‐5272 (−)

Because both cisplatin and DS‐5272 are anticancer treatment that may cause bone marrow suppression, we investigated the toxicity of DS‐5272 on bone marrow function. In the control model without cisplatin, DS‐5272 induced neutropenia, but not anemia nor thrombocytopenia on day 10 (cisplatin(−)/DS‐5272(−) vs cisplatin(−)/DS‐5272(+)) (Figure 6). Ten days after cisplatin injection, the vehicle group had developed neutropenia and thrombocytopenia compared to that of the control group (cisplatin(−)/DS‐5272(−) vs cisplatin(+)/DS‐5272(−)) (Figure 6). The DS‐5272 group also showed neutropenia and thrombocytopenia but the extent of both did not differ between the two cisplatin groups (cisplatin(+)/DS‐5272(−) vs cisplatin(+)/DS‐5272(+)) (Figure 6). Therefore, DS‐5272 and cisplatin co‐treatment does not elicit additive or synergistic bone marrow toxicity.

## 4 DISCUSSION

In this study, we investigated the effect of the MDM2 inhibitor, DS‐5272, on cisplatin‐induced kidney injury and the mechanism involved. The results demonstrated that cisplatin caused structural and functional kidney injury in association with increased NFκB activation and that DS‐5272 ameliorated these cisplatin‐induced kidney injuries and suppressed NFκB phosphorylation. In cultured cells, DS‐5272 decreased cisplatin‐induced cell death and inhibited NFκB transcriptional activity.

We had previously shown that Nutlin‐3a, another MDM2 inhibitor, has protective effects in postischemic acute kidney injury 12 and crescentic glomerulonephritis 13 via p53‐independent NFκB signal inhibition. NFκB, which is a central mediator of cisplatin nephropathy, leads to proinflammatory cytokine transcription in this disease.^10^, ^16^ It has been reported that inhibition of NFκB signaling has protective effects on cisplatin nephropathy.^20^, ^21^, ^22^, ^23^, ^24^ Our data indicated that DS‐5272 inhibits NFκB signaling, which contributes to amelioration of cisplatin nephropathy in vivo and decreased cell injury in vitro. In this study, however, there were two major discrepancies between the time course of MDM2 expression and the effect of DS‐5272. First, MDM2 and phospho‐MDM2 were not altered on day 3, when the effect of DS‐5272 was observed (Figure 3). In our previous ischemic kidney injury study, after Nutlin‐3a treatment, neither MDM2 nor phospho‐MDM2 was changed at the kidney injury phase.^12^ Thus, we considered that Nutlin‐3a acts as a cofactor for NFκB at target gene promoters without changing MDM2 expression.^2^, ^12^ In addition, MDM2 directly activates NFκB p65 mRNA transcription by interacting with Sp‐1 biding site in the p65 gene promoter region in leukemia cells.^25^ We, therefore, speculate that DS‐5272 inhibits NFκB p65 transcription not by degrading MDM2 protein, but by inactivating NFκB transcription. Second, MDM2 and phospho‐MDM2 level began to increase by day 5 after cisplatin injection (Figure 1) although the effect of DS‐5272 was seen by day 3 (Figure 3). Cisplatin treatment induces p53 phosphorylation and p53 expression in vivo^26^ and in vitro.^27^ Since p53 and MDM2 are known to regulate each other via a negative feedback loop, p53 activation leads to expression of MDM2 expression.^28^, ^29^ Thus, increased MDM2 protein by day 5 in this study may be simply the result of cisplatin‐induced kidney injury.

DS‐5272 had protective effects only on day 3, and not on days 5 or 10 (Figure 2). Thus, it is uncertain whether DS‐5272 has any effect on disease progression. However, a human observational study had shown previously that an increase in serum creatinine of only 0.3 mg/dL or more was associated with significantly greater mortality.^30^ Furthermore, in a human systematic review, acute kidney injury was associated with greater long‐term mortality and higher incidence of chronic kidney disease, even after recovery from acute kidney injury.^31^ Therefore, ameliorating the early‐phase of kidney injury is considered worthwhile, despite the absence of significant differences on days 5 and 10.

While many experimental studies have been performed to develop a therapy that can prevent or treat cisplatin nephropathy, the only protective therapy currently implemented in clinical practice is hydration.^32^ Specific renoprotective interventions that do not interfere with the cisplatin‐related antitumor effects need to be developed. Because MDM2 inhibitors were also developed as anticancer drugs and have been reported to possess anticancer effects in animal models, the use of MDM2 inhibitors seems intriguing in this context. Human preliminary trials have confirmed their efficacy and tolerability.^33^, ^34^, ^35^ Considering these studies and our data, MDM2 inhibition may be a promising strategy to attenuate cisplatin nephrotoxicity and synergistically supports its antitumor effects with an acceptable safety profile.

Several limitations of this study should still be mentioned. First, the results of this study demonstrated no direct evidence that DS‐5272 mediated its protective effect through MDM2 inhibition. In our previous ischemic kidney injury study, although MDM2 protein expression was not changed at the injury phase after Nutlin‐3a treatment, MDM2‐deficient mouse embryonic fibroblasts were used in order to verify the necessity of MDM2 for the Nutlin‐3a effect.^12^ Second, the experimental group size appears to be too small to fully establish the validity of the protective effect of DS‐5272. A future study, designed to explore the detailed mechanism with a larger group size, is needed.

In summary, our data demonstrated that DS‐5272 ameliorated cisplatin‐induced kidney injury in vivo and decreases cisplatin‐induced cell injury in vitro via NFκB inhibition. As this MDM2 inhibitor exhibits anticancer effects, its usage is a promising strategy to prevent cisplatin‐induced kidney injury in the future.

## AUTHOR CONTRIBUTIONS

T.F. and Y.H. participated in research design. T.F., I.T., and T.T conducted experiments. T.F., Y.H. and H.J.A. wrote or contributed to the writing of the manuscript.

## DISCLOSURES

None declared.
